# Perspectives of French adolescents with ADHD and child and adolescent psychiatrists regarding methylphenidate use

**DOI:** 10.1038/s41598-023-30921-4

**Published:** 2023-03-10

**Authors:** Jordan Sibeoni, Emilie Manolios, Clement Hausser, Raphael Delage, Franck Baylé, Mario Speranza, Laurence Verneuil, Anne Revah-Levy

**Affiliations:** 1 Service Universitaire de Psychiatrie de l’Adolescent, Argenteuil Hospital Centre, 69 rue du Ltc Prud’hon, 95100 Argenteuil, France; 2 ECSTRRA Team, UMR-1153, Inserm, Université Paris Cité, 75010 Paris, France; 3 APHP, Service de psychiatrie et addictologie de l’adulte et du sujet âgé, hôpital européen Georges Pompidou, 75015 Paris, France; 4 GHU Paris psychiatrie et neurosciences Site Sainte-Anne, 75014 Paris, France; 5 Service Universitaire de Psychiatrie de l’Enfant et de l’Adolescent, Centre Hospitalier de Versailles, 177 Rue de Versailles, 78150 Le Chesnay-Rocquencour, France; 6 Centre de Recherche en Épidémiologie et Santé des Populations (CESP), INSERM UMR 1018 «Developmental Psychiatry and Trajectories», Université Paris-Saclay, Université Versailles Saint Quentin en Yvelines, 16 Av. Paul Vaillant Couturier, 94800 Villejuif, France; 7 Pôle Précarité, GHU Paris psychiatrie et neurosciences Site Sainte-Anne, 75014 Paris, France

**Keywords:** Psychology, Outcomes research, Paediatric research

## Abstract

Many studies have demonstrated the short-term efficacy and tolerability of methylphenidate treatment adolescents with attention deficit hyperactivity disorder (ADHD). Qualitative literature on this matter focused on school outcomes, long-term side effects, family conflicts, personality changes and stigmatization. Yet, no qualitative study has crossed the perspectives of child and adolescent psychiatrists (CAPs) prescribing methylphenidate and adolescents with ADHD. This French qualitative study followed the five stages IPSE—Inductive Process to analyze the Structure of lived Experience-approach. Fifteen adolescents with ADHD and 11 CAPs were interviewed. Data collection by *purposive sampling* continued until data saturation was reached. Data analysis, based on a descriptive and structuring procedure to determine the structure of lived experience characterized by the central axes of experience, produced two axes: (1) The process of methylphenidate prescription, highlighting how this prescription was motivated from the exterior, experienced as passive by the adolescents and required commitment from the CAPs; and (2) the perceived effects of methylphenidate treatment, in three domains: at school, in relationships and in the sense of self. Findings raised both the issues of the epistemic position and social representation of the adolescents about ADHD and methylphenidate within this specific French context, and the self-awareness and perception of the adolescents with ADHD. We conclude that these two issues need to be regularly addressed by the CAPs prescribing methylphenidate to avoid epistemic injustice and prevent the harmful effects of stigmatization.

## Introduction

Attention deficit hyperactivity disorder (ADHD) is characterized by the presence of developmentally inappropriate levels of hyperactive-impulsive and/or inattentive symptoms for at least 6 months in different settings and that impair various aspects of the patient's life^1^, the onset of symptoms and impairments occurring before age 12^2^. Poor outcomes have been described, especially in the absence of treatment, including poor quality of life, substance use disorders, accidental injuries, educational underachievement, unemployment, difficulties socializing, delinquency, suicide, and premature death^3^. ADHD is one of the most researched disorders in psychiatry in many fields^3^ including research about genetic causes, environmental correlates, neuropsychological and cognitive processes, and co-occurrence with other psychiatric disorders^4,5^, but also social sciences research focusing on the epistemic validity of ADHD and on the ethical and sociological implications of its treatment^6^.

It is the most frequent neurodevelopmental disorder in children^3^. Community prevalence has been estimated between 2 and 7%^7^. In their meta-regression analysis pooling data from 135 original studies, Polanczyck et al.^8^ found that neither the geographical location nor the year of study were associated with variability in the estimation of the prevalence. However, a large cohort study conducted in Israel reported an increase in the ADHD prevalence rate from 6.6 to 14.4% between 2005 and 2014^9^, while in France, one study has estimated the prevalence of ADHD for French children aged 6–12 at from 3.5 to 5.6%^10^ and, a more recent one^11^, using data from French health care insurance reimbursements for methylphenidate prescription, estimated the prevalence rate of ADHD for French children aged 6–11 at only 0.3%. ADHD epidemiology is a controversial question^12^, especially in France. Commenting on the study of Ponnou and Haliday^10^, Ramus and Peyre^13^ pointed out its methodological flaws, especially the fact that “in France, methylphenidate is not the first-intention treatment for ADHD.” Indeed, the French guidelines, from the Haute Autorité de Santé (High Autority of Health) state that for children and adolescents “in the first instance, non-pharmacological interventions should be implemented, combining psychological, educational and social measures according to the child's needs. If these measures are insufficient, medication may be initiated (…) and must be integrated into a personalized approach for each child, reevaluated every month and prescribed in addition to non-pharmacological interventions.”^14^.

In France, *Methylphenidate* (*MPH*) is the only medication available today that is indicated as pharmacological treatment of ADHD^14^. Prescribing rules are strict, only psychiatrists, child and adolescent psychiatrists (CAPs), neurologists, and pediatricians are allowed to diagnose ADHD (or to confirm the pre-diagnosis of first-lines doctors), to initiate such treatment and make the annual renewal. Other renewals, every 28 days can be done by any physician^14^. In France, access to such specialists is limited, especially for CAPs and in some rural or poor areas, resulting a delay in diagnosing and treating ADHD. Moreover, assessment is quite heterogenous among French CAPs, gradually (1) a sole clinical assessment, (2) the extra use of functional subjective scales completed by parents and teachers, mainly the ADHD RS^15^ and the Conners^16^, and (3) neuropsychological assessments including a cognitive evaluation and attentional tests such as the Test of everyday attention for children (TEA-Ch). Many non-pharmacological treatments (such as cognitive-behavioral therapies, neurofeedback, positive Parenting Program, or parent–child interaction therapy) are still not widely available. Five methylphenidate drugs are available in France (Ritaline^®^, Ritaline LP^®^, Quasym^®^, Concerta^®^, and Medikinet^®^ differing in terms of release rates). Since its initial marketing authorization in 1996, consumption—initially low—has grown significantly and constantly^17^, with a major acceleration in sales in 2004, concomitant with the introduction of the extended-release drug forms. The consumption among adolescent increased by 21.5% between 2012 and 2014, French adolescents the latter year accounted for about 40% of the persons receiving this treatment. Methylphenidate use nonetheless remains relatively low in France compared to other developed countries^17^. This echoes a lack of consensus regarding how to consider and approach ADHD in France with different opinions and views, not only in the French society^18^ but also among French mental health professionals. As a matter of fact, there is an important heterogeneity of practice among French CAPs for using the concept of ADHD and using methylphenidate as a treatment.

Many randomized controlled trials have shown the short-term efficacy and tolerability of methylphenidate for ADHD^3^. A comprehensive network meta-analysis of 133 double-blind RCTs demonstrated its short-term efficacy versus placebo and showed that it was the only drug with better acceptability than placebo^19^. Systematic reviews have shown that psychostimulant treatment is often associated with better outcomes in adolescence: better academic performance and social relationships and fewer accidents^20^. There are however some concerns about side effects of medications—including appetite suppression, sleep issues, and impact on growth with extended treatment^21^—as well as a lack of evidence about its long-term efficacy.

Adolescence is a complex developmental period with identity and relational turmoil that can impact both clinical aspects of ADHD and treatment. The course of ADHD varies highly through adolescence with different trajectories being modelized in the literature from age-related improvement—more for hyperactivity-impulsivity symptoms than attention deficit—to worsening symptoms^22^. ADHD also acts a risk factor for the emergence of many disorders during adolescence, especially substance use and mood disorders^23^. ADHD during adolescence is a major predictor of an array of physical, mental, work, and financial problems in adulthood^24^. Furthermore, specific issues related with adolescence—identity construction, intersubjective and social relations—collide with ADHD. Several qualitative studies have emphasized that adolescents experience and understand ADHD through the core aspects of self (self-image, self-concept, self-stigma), identity development, agency, and social connectedness^25–28^. Some social factors, such as social acceptance, have showed some protective effects on academic functioning on adolescents with ADHD^29^. A metasynthesis of 11 qualitative studies exploring the lived experience of adolescents diagnosed with ADHD found similar themes—societal pressure, sense of self—but also maturational shift from passive to active and feelings about medication^30^. Indeed, specific issues have been described regarding the use of stimulants among adolescents with ADHD. Buitelaar^31^ argued that therapeutic strategies in adolescents with ADHD are merely the same as the one for children and are not targeting specific aspects that make “*adolescents special and vulnerable, such as poor insight into own functioning and poor decision making*”. Poor medication adherence^31^, treatment discontinuation^32^ and misuse^33^ of methylphenidate among adolescents with ADHD have been reported.

There is already a consistent qualitative literature exploring the views about methylphenidate treatment for adolescents with ADHD, coming mostly from Western English-speaking countries and collecting data from adolescents or young adults with ADHD^28,34–37^, their families^28,34,35,38–41^ healthcare professionals—both first-line and specialists^35,38,39,42^ and teachers^35,39^, through individual interviews or focus groups. The findings report some positive outcomes—fewer problems at home and at school, reduction of disruptive behavior, improvement of peer relationships^37,39^—and some negative experiences with methylphenidate use, such as side effects^28,35,36^, changes in sense of self^28,34,37^ and stigma—yet more related with the ADHD symptoms than the medication use^37^. Several studies address how difficult the decision to use medication was for the parents^34,40^ while Leslie et al.^41^ described different patterns in which parents first either chose non-pharmacological treatment or rapidly engaged in the medication use. Two studies reported, among adolescents and parents, the progressive autonomy and transfer of responsibility for managing medication^28,34^. The issue of continuation or cessation of treatment, along with breaks through planned holidays—was the focus of three qualitative studies among healthcare professionals^38,39,42^, reporting how some patients could manage to stop the treatment, while other “felt the need to restart”^39^.

To our knowledge, no qualitative study has yet examined this specific topic in France.

The aim of our study was therefore to explore the lived experience of methylphenidate use among French adolescents with ADHD and child and adolescent psychiatrists (CAPs) and to cross the perspectives of these two groups so to develop original insights.

## Methods

This exploratory qualitative study used the Inductive Process to analyze the Structure of lived Experience (IPSE) approach^43^. IPSE is informed by a descriptive phenomenological approach and relies on an inductive process that probes in depth the lived experience of patients and healthcare professionals and analyzes the structure of their experiences. Five stages, described below, structure the research process. The report of this study adheres to the COREQ guidelines^44^ (supplemental material S1). All procedures involving patients and professionals were:

(i) approved by the "Conseil pour l'évaluation ethique des recherches en santé Université Paris-Déscartes" (the University of Paris-Descartes council for the evaluation of ethics in health research). All adolescents and their parents provided written informed consent before inclusion in the study.
(ii) performed with relevant guidelines—COREQ^44^—and regulations, that is in accordance with the declaration of Helsinki regarding the principles for medical research involving human subjects.

### Stage 1: Setting up a research group

Our research group included four psychiatrists, each with different experience regarding ADHD and methylphenidate prescriptions: one has never prescribed methylphenidate, one prescribes it occasionally, and two specialists in ADHD, prescribing it regularly. The group also included two psychiatric trainees, a psychologist, and a medical doctor from another specialty, all trained in qualitative methods. For heuristic purposes—that is, to enable to discover new unknown elements and to produce original findings—the group’s members were highly diverse, especially in their knowledge, age, and backgrounds. The group worked continuously on reflexivity during open discussions among themselves.

### Stage 2: Ensuring the originality of the study

Two members of the group reviewed the qualitative and quantitative literature systematically, to confirm the study's relevance and originality. They verified that no qualitative study of this specific topic and explicitly crossing the perspectives of adolescents and CAPs had been conducted. To ensure that the other group members could remain inductive and open to novelty, they had access to this review only after they had completed the data analysis.

### Stage 3: Recruitment and sampling, aiming for exemplarity

The research group defined the inclusion and exclusion criteria (Table 1) intended to attain exemplarity, that is, to select participants who “have experienced quintessential, typical, or archetypal examples of the situation being studied”^43^ and to include participants who might enrich and add something new to previous findings. We thus used a purposive sampling strategy with maximum variation^45^, that is to select adolescents who differed by sex, age, family status, duration of disease and methylphenidate treatment, and outcomes of care; and to select CAPs who differed by sex, age, frequency of methylphenidate prescription, and work setting. Researchers identified potential participants whom they considered likely to provide the most information. In practice, after presenting the study design and inclusion criteria during an initial meeting, the research group had regular exchanges with the physicians, during the data collection period, in both child-adolescent psychiatry departments in which recruitment took place so to ensure a variation of those criteria and a variety of exemplary situations. Thus, the physicians helped us to identify adolescents that had relevant and original information to provide.

**Table 1:** Inclusion and exclusion criteria.

	Adolescents	Child and adolescent psychiatrists
Inclusion criteria	Age: 12–18	Currently practicing
	Diagnosis of ADHD (according to DSM-5) as the principal diagnosis	
	Methylphenidate treatment	Prescribed methylphenidate (initiation or renewal) to at least one adolescent in the past 12 months
	At the time of the interview	
	Initiated at least 3 months earlier	
	Monotherapy or associated with melatonin	
Exclusion criteria	Psychiatric comorbidities such as schizophrenia or autism spectrum disorder	
	Intellectual disabilities	
	Acute symptoms at inclusion	
	Treatment by another psychotropic medication (mood stabilizer, antidepressant, antipsychotic)	

*DSM*-5 The Diagnostic and Statistical Manual of Mental Disorders, Fifth Edition, *MDD* major depressive disorder.

Sample size was not defined in advance but was determined by data saturation according to the principles of “information power”^46^—here based on the criteria described by the authors: “the quality of dialogue” during the interview, “the aim of the study” and the “sample specificity”, that is “the specificity of experiences, knowledge, or properties among the participants included in the sample”^46^. For both subgroups, inclusion of new participants continued until the analysis of new material no longer yielded new findings; that is, data collection and analysis were complete when the research group considered that the axes of experience obtained provided a sufficient explanatory framework for the data collected^47^.

Patient recruitment took place in two child-adolescent psychiatry departments in the Paris suburbs during face-to-face encounters; they had catchment areas with distinctively different socioeconomic profiles. CAPs were recruited in both public hospitals and private practice, from the networks of both departments through emails and telephone.

### Stage 4: Data collection, access to experience

One-to-one interviews were conducted in the psychiatric department for the adolescents and at the workplaces for the CAPs by two male psychiatry trainees (RD, CH), and one female psychologist (EM), all public health researchers (introduced as such to the research participants) trained in qualitative methods. Interviewers did not have any contact with the participants prior to the study. They conducted semi-structured interviews using an *open-ended approach*^48^, structured by areas of exploration (Table 2) collectively determined by the group, from listening and reading two pilot interviews, neither included in the final data. The interviews lasted 50–70 min. They were audio-recorded and transcribed into anonymized transcripts, including the participants' expressive nuances.

**Table 2:** Interview guide.

Area of exploration	Potential questions (adolescent)	Potential questions (psychiatrist)
1. Initiating/renewing/stopping methylphenidate treatment	Can you tell me what led to this methylphenidate treatment? Can you tell me what happened during the last consultation when your doctor prescribed the medication?	Can you tell me what happened during the last consultation you prescribed methylphenidate to a teenager? How did the parents react to this prescription?
2. Treatment options and decision-sharing	How was the decision made? What were the other options?	How do you make the decision to prescribe methylphenidate? What were the other options?
3. Knowledge, literacy	What do you know about methylphenidate? How do you feel about the fact that you are taking methylphenidate?	How do you present this treatment and its action to patients and parents?
4. Outcomes/effects	How do you feel when taking methylphenidate? How does it affect you?	What effects did you observe? What are the most relevant outcomes your patients and their families tell you about?
5. Issues/obstacles	What issues did you encounter?	What kind of issues do adolescents and their parents raise during consultations?
6. Global treatment plan	What about other therapeutic interventions?	How do you integrate methylphenidate treatment with other therapeutic interventions?

The guide was translated from French to English for the sole purpose of this article.

### Stage 5: Data analysis, from the description of the structure of experience to practical implications

The analytic procedure followed the IPSE approach^43^. The IPSE analytic process is a rigorous procedure that relies on an inductive, phenomenological method^43^. In practice, the analysis had two stages: one stage of independent work by the three researchers, aided by Nvivo software, and the other by the group as a whole, pooling the data collectively. In the individual procedure, the three qualitative researchers independently and simultaneously conducted a systematic descriptive analysis to convey each participant’s experience. This involved for each interview: (1) listening to the recorded interview twice and reading it three times; (2) exploring the experience word by word, that is, cutting up the entire text into descriptive units; (3) regrouping the descriptive units into categories. These stages are carried out with the help of QSR NVivo 12 software. During the group process, the three researchers met regularly with the other group members, who had familiarized themselves with the data by listening to and reading all the interviews as many times as necessary. These two-hour meetings began after the analysis of five interviews. The first set was intended to conduct the structuring phase, that is, to regroup the categories into axes of experience, constructed such that each could be linked to its subjacent categories, and then to determine the structure of lived experience characterized by the central axes. The second set of meetings covered the practical phase, the process of triangulation with the data in the literature that made it possible to identify the original aspects of the results and to suggest potential practical implications of the finding to improve care.

We used several criteria to ensure the rigor of the analysis and the trustworthiness of the results: triangulation, attention to negative cases, reflexivity within the group process, and feedback from "subjects of the experience” by presenting the results to a group of adolescents with ADHD and treated with methylphenidate (N = 5) and a group of CAPs who had experience using methylphenidate to treat adolescents with ADHD (N = 5). They all recognized their own experience in the structure we proposed.

Reflexivity—the researchers’ reflection of their role in the study and its effects on their findings at every step of the research process—was worked on constantly in the group, during open discussions between the researchers. To address the diversity of reflexive positions within the group, two questions were asked to each member: (i) “What are your preconceptions and my beliefs about the phenomenon under study and the research question? “And (ii) “What are your expectations regarding this study?”. All the members listed and shared with the group their preconceptions and beliefs about both ADHD and methylphenidate treatment, as well as their personal motives to be part of this research and what they were expecting to find or achieve.

## Findings

The study included 15 adolescents (4 girls and 11 boys) and 11 CAPs specialized in treating adolescents for a total of 26 participants. No one approached refused to participate.

Table 3 summarizes the adolescents’ characteristics. All the adolescents recruited agreed to participate. Their ages ranged from 12 to 18 years and averaged 14.8 years. Nine adolescents were diagnosed with ADHD during childhood (i.e., before the age of 12). Duration of methylphenidate treatment ranged from 1 to 10 years; its mean duration was 3.7 years. All of them received a modified- or extended-release form of methylphenidate either alone or in association with an immediate-release component. Table 4 summarizes the CAPs’ characteristics (5 women, mean age 39.7 years). Three worked part- or full-time in ADHD units. Most worked in the Paris metropolitan area, the French region with the highest population density and socioeconomic heterogeneity.

**Table 3:** Adolescents’ characteristics.

Adolescent (A)	Sex	Age	Psychiatric comorbidities	Parental socio-economic-category	Age at ADHD diagnosis	Duration of methylphenidate treatment (years)	Current treatment
A1	M	13		Middle	10	4	Concerta LP 36 mg 5 days a week
A2	F	16	Specific learning disorder (reading, written expression)	High	13	2	Quasym LP 40 mg 7 days a week
A3	F	12		Middle	9	3	Ritalin LP 20 mg + ritalin 10 mg 5 days/week
A4	M	14		High	12	2	Concerta LP 36 mg 5 days/week + melatonin 2 mg
A5	M	17	Social anxiety	Middle	6	6	Ritalin LP 30 mg 5 days/week + melatonin 2 mg
A6	M	12	Specific learning disorder (reading, written expression)	Low	10	1	Ritalin LP 20 mg, 5 days/week
A7	M	17		Middle	7	8	Medikinet 40 mg 7 days/week
A8	M	18	General anxiety disorder	High	16	1	Concerta LP 54 mg 5 days/week
A9	M	15	Specific learning disorder (written expression)	Middle	8	6	Quasym LP 30 mg 5 days/week + melatonin 2 mg
A 10	F	15		Middle	11	3	Ritalin LP 30 mg 5 days/week
A11	M	16		Middle	13	3	Concerta LP 54 mg 5 days/week
A12	F	14		Middle	7	7	Quasym LP 30 mg 5 day/week
A 13	M	13		Middle	9	6	Concerta LP 36 mg 5 days/week
A 14	M	15	Language disorder	Middle	10	4	Medikinet 20 mg 5 days/week
A 15	M	16		Low	9	7	Ritalin LP 30 mg 5 days/week

**Table 4:** CAPs’ characteristics.

CAPs	Age	Sex	Patients under methylphenidate treatment (%)—self reported	Status	Work setting	Specific training	Country of training
P1	51	M	15	Hospital staff physician	Outpatient unit	Mentalization-based therapy	France
P2	41	F	80	Hospital staff physician	ADHD day hospital	Neurosciences (MSc), Mentalization-based therapy	Portugal
P3	30	F	10	Junior physician	Inpatient unit	Family therapy	France
P4	41	F	10	Hospital staff physician	Inpatient unit and liaison	Family therapy	France
P5	33	M	30	Chief resident	Specialized inpatient + outpatient units	Neuropsychology, Neurosciences (MSc)	France
P6	31	M	25	Senior physician	Learning disorder day hospital + outpatient unit	Neurosciences (MSc)	France
P7	59	M	50	Professor	Outpatient unit	Epidemiology and statistics (PhD)	France
P8	35	M	10	Chief resident	Inpatient unit + Learning disorder private practice	Cognitive science (MSc), epidemiology and statistics	France
P9	35	F	5	Hospital staff physician	Inpatient unit	Family therapy, hypnosis	France
P10	34	M	25	Senior physician	Private practice + autism spectrum disorder unit		France
P11	47	F	30	Hospital staff physician	Outpatient unit	Cognitive behavioral therapy, hypnosis	France

**Figure 1:**
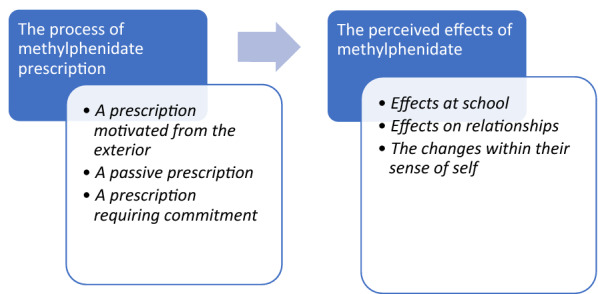
Diagram of the findings.

Data analysis produced a common structure of lived experience based on two central axes of experience: (1) the process of methylphenidate prescription, and (2) the perceived effects of methylphenidate (Fig. 1). Transcript excerpts presented below have been selected to exemplify the themes described and translated into English by a professional scientific translator for the sole purpose of this article. The original excerpts, in French, can be found in Table 5.

**Table 5:** Original French quotations.

**The process of methylphenidate prescription**
*A prescription motivated from the exterior*
A1 No, it was the school who said to my parents that I was too scatterbrained in class, (…) I was a child, children they are joyful and have fun, no? **Non, c'est l'école qui a dit à mes parents que j'étais trop *foufou* en classe, (…) j'étais un enfant, les enfants ils sont joyeux, ils s'amusent, non ?**
A11 So I started Concerta in year 7 (7th grade) because they said I had trouble getting myself to start working, problems of organization ** J'ai donc commencé le Concerta en 5**^**ème**^** parce qu'ils disaient que j'avais du mal à me mettre au travail, des problèmes pour m’organiser**
A10 My mother always wanted me to do my homework, extra homework, or extra exercises, and I didn't want to, so it created problems at home… The ambiance at home was not good ** Ma mère voulait toujours que je fasse mes devoirs, des devoirs en plus ou des exercices en plus, et je voulais pas, alors ça créait des problèmes à la maison… L'ambiance à la maison elle était pas bonne**
A8 …the teachers said there was something wrong with me … they said I had a little problem and that there was a medication that might help ** …les professeurs ont dit que quelque chose n'allait pas chez moi… ils ont dit que j'avais un petit problème et qu'il y avait un médicament qui pouvait m'aider**
A4 I didn’t know what it was to be hyperactive. I thought I was just a normal child who had stronger adrenaline ** Je ne savais pas ce que c'était d'être hyperactif. Je pensais que j'étais juste un enfant normal qui avait une poussée d’adrénaline**
P9: Parents especially can’t stand it anymore, it's 'it's unbearable, give him a good dose’ ** Les parents surtout n'en peuvent plus, c'est " c'est insupportable, donnez-lui une bonne dose "**
P6 …the school sent an ultimatum: if it's not treated rapidly, he'll have to repeat the year …**l'école lui a posé un ultimatum : s'il n'est pas traité rapidement, il redoublera**
*A passive prescription*
A15 I was passive. … my parents, they are really very stubborn. They profoundly believe that if there's a medication to take, you buy it, and you take it ** J'étais passive. … mes parents, ils sont vraiment très têtus. Ils croient profondément que s'il y a un médicament à prendre, on l'achète et on le prend**
A5 At the beginning I didn't want to take it.. But afterwards, my parents sort of forced me … my father told me, "try it and then you'll see yourself if you want it’. I saw it was really not bad at all, I continued taking it … not so much for my grades. OK for my parents it's for my grades, for me it helped me in my everyday life, ** Au début, je ne voulais pas le prendre. Mais après, mes parents m'ont un peu forcé… mon père m'a dit : " essaie et puis tu verras par toi-même si tu en veux ". J'ai vu que c'était vraiment pas mal du tout, j'ai continué à en prendre … pas tellement pour mes notes. OK pour mes parents c'est pour mes notes, pour moi c’est plus pour m'aider dans la vie de tous les jours,**
P8 They were truly led here by their parents. If you asked them, they had no reason to be here ** Ils ont vraiment été amenés ici par leurs parents. Si vous leur demandez, ils n'avaient aucune raison d'être ici**
*A prescription requiring commitment*
P10 … because inevitably when you think it's indicated, you push a little to prescribe it ** parce qu'inévitablement, quand on pense que c'est indiqué, on pousse un peu pour le prescrire**
P7 I prescribe medication for children who are ill. I do not prescribe drugs for children to have the best grades in school, it's an ethical question ** Je prescris des médicaments aux enfants qui sont malades. Je ne prescris pas de médicaments aux enfants pour qu'ils aient les meilleures notes à l'école, c'est une question d'éthique**
**The perceived effects of methylphenidate**
*Effects at school*
A14 (Do you take the medication on the weekend too?) Only when I have to work ** (Prenez-vous aussi vos médicaments le week-end ?) Seulement quand je dois travailler**
A2: I don't think I'll take it for my whole life, but today I think that without it I would not necessarily manage to meet the objectives I've set for myself ** Je ne pense pas que je le prendrai toute ma vie, mais aujourd'hui je pense que sans lui, je n'arriverais pas forcément à atteindre les objectifs que je me suis fixé**
A8: I don't know if it’s doping or not … the person still has to make an effort … without the work, it wouldn't accomplish anything. It's not going to make someone intelligent ** Je ne sais pas si c'est du dopage ou pas… la personne doit quand même faire un effort… sans le travail, ça ne servirait à rien. Ça ne va pas rendre quelqu'un intelligent**
P7: … he's different so society, school, don't tolerate him; so we make it possible for him to be tolerated ** il est différent donc la société, l'école, ne le tolèrent pas ; donc nous faisons en sorte qu'il soit toléré**
*Effects on relationships*
A2: I noted that I was fighting less, I was becoming more friendly with people ** J'ai remarqué que je me battais moins, que j’étais plus sympa avec les gens**
A15 "I noticed that it was cutting off my relationships with others.” ** J'ai remarqué que ça me coupait de mes relations avec les autres**
P7 … the teachers also have a representation, and as a result they say to themselves, no it's not that he doesn't care about anything, in fact, he's different ** … les enseignants ont aussi une représentation, et du coup ils se disent, non c'est pas qu'il se fout de tout, en fait il est différent**
*The changes within their sense of self*
A9: Before, I was extremely dreamy, that is, I always had a story in my head, that I was inventing, like that … but that was before ** Avant, j'étais extrêmement rêveur, en fait j'avais toujours une histoire dans la tête, que j'inventais, comme ça… mais c'était avant**
A14: I don't know if it's a medication or a form of torture, except for tests at school (…), the feeling of malaise that I can feel sometimes, it's torture … I'm someone who is well connected to my feelings, but it reconfigures me ** Je sais pas si c'est un médicament ou une forme de torture, sauf pour les exams à l'école (…), le sentiment de malaise que je peux ressentir parfois, c'est une torture… Je suis quelqu'un qui est bien connecté à mes sentiments, mais ça me reconfigure**

### The process of methylphenidate prescription

#### A prescription motivated from the exterior

Adolescents and CAPs reported that the process leading to methylphenidate treatment originated with the identification of problems in the exterior world—in the family or in a school setting—but that the adolescents did not appear to perceive internally.

*A1 *“*No, it was the school who said to my parents that I was too scatterbrained in class, (…) I was a child, children they are joyful and have fun, no*?”

School problems concerned schoolwork—difficulties in learning and in concentration, poor grades—as well as inappropriate behavior in school. Some adolescents experienced painfully the fact of “being bad at school” and reported being portrayed as “dumb” (A2) or “stupid” (A4) by their peers and even their teachers.

*A11* “*So I started Concerta in year 7 (7th grade) because they said I had trouble getting myself to start working, problems of organization*.”

The family problems concerned conflicts around school results and schoolwork, or relationship problems with the parents or siblings.

*A10 *“*My mother always wanted me to do my homework, extra homework, or extra exercises, and I didn*'*t want to, so it created problems at home… The ambiance at home was not good.*”

The adolescents reported that their parents, and sometimes their teachers, attributed all the problems to ADHD and leading to the search for professional help, sometimes directly for a methylphenidate prescription.

*A8 *“*the teachers said there was something wrong with me … they said I had a little problem and that there was a medication that might help*”.

Most of the adolescents did not recognize themselves in this process and mentioned a discrepancy between their perception of their problems and the attribution to a disorder. They did not consider these "problems" to be pathologies, but rather perceived them as part of their identity. They described themselves as "normal" (A5) and used expressions such as "head in the clouds" (A2) and "scatterbrained"(A1). Only some adolescents retrospectively recognized an abnormal aspect to their experience, but only in comparison with other children.

*A4 *“*I didn*’*t know what it was to be hyperactive. I thought I was just a normal child who had stronger adrenaline*.”

The CAPs all reported that this request came from families or school based on externalized problems. Some CAPs underlined the parents' exhaustion or their high demands of the child.

*P9: Parents especially can*’*t stand it anymore, it*'*s *'*it*'*s unbearable, give him a good dose*’*.*

Others recognized the need to respond to a demand expressed above all by the school.

*P6 *“*the school sent an ultimatum: if it*'*s not treated rapidly, he*'*ll have to repeat the year.*”

#### A passive prescription

The adolescents described themselves as passive in the decisions related to the start of medical treatment. They said they "obeyed" (A2) or "complied with" (A14) the decisions of adults in general and of their parents in particular.

*A15″ I was passive. … my parents, they are really very stubborn. They profoundly believe that if there*'*s a medication to take, you buy it, and you take it.*”

Some had initially refused the treatment, because that would have confirmed the abnormal nature of their behavior. Once they tried the treatment, most were convinced of its interest but for reasons different from those of their parents.

*A5 *“*At the beginning I didn*'*t want to take it (…) my father told me, "try it and then you*'*ll see yourself if you want it*’*. I saw it was really not bad at all, I continued taking it … not so much for my grades. OK for my parents it*'*s for my grades, for me it helped me in my everyday life,*”

The CAPs also reported some *passive* prescription situations, where parents had "almost dictated the Ritalin prescription to me (P2)." Some mentioned an erosion of their medical role, reduced to the sole act of writing the prescription. They also perceived the passivity of the teen, who seem to them to be led by his or her parents and who had no requests of his or her own.

*P8 *“*They were truly led here by their parents. If you asked them, they had no reason to be here*”*.*

#### A prescription requiring commitment

The CAPs reported few situations where the family was not convinced that a treatment by methylphenidate was necessary. The CAPs explained that they didn’t hesitate to be more insistent and directive, especially when they were sure the adolescent would benefit from this treatment.

*P10 *“*because inevitably when you think it*'*s indicated, you push a little to prescribe it.*”

The CAPs reported the current situation in France regarding the lack of consensus between psychiatrists about to the use of methylphenidate and the views about ADHD. Prescribing methylphenidate meant also taking a personal "activist" stance (P1), *choosing a camp* within the discipline of child and adolescent psychiatry, and supporting "the theoretical point of view" (P8) that ADHD is a neurodevelopmental disorder.

They were aware that some people (both from the society and the discipline) could consider their prescription as “biological empowerment” and highlighted their medical professionalism and ethics.

*P7 *“*I prescribe medication for children who are ill. I do not prescribe drugs for children to have the best grades in school, it*'*s an ethical question.*”

### The perceived effects of methylphenidate

Adolescents and CAPs principally reported effects on the outside world—school, but also relationships and, to a lesser extent, on the adolescents themselves.

#### Effects at school

The adolescents reported first of all numerous changes related to school: better attention and concentration in class, faster note-taking, better quality writing, and a greater capacity to work. They insisted principally on two points: increased autonomy and better performance at school. These numerous benefits at school led the adolescents to consider the drug as a tool for their schoolwork, adapting their intake solely according to this variable.

*A14 *“*(Do you take the medication on the weekend too?) Only when I have to work*”

Many adolescents saw in this drug a guarantee of their academic and professional success. They associated their academic and future professional objectives with continuation of this treatment.

*A2: *“*I don*'*t think I*'*ll take it for my whole life, but today I think that without it I would not necessarily manage to meet the objectives I*'*ve set for myself.*”

Finally, they asked themselves about the doping nature of the product.

A8: “*I don*'*t know if it*’*s doping or not … the person still has to make an effort … without the work, it wouldn*'*t accomplish anything. It*'*s not going to make someone intelligent.*”

Some psychiatrists also reported questions by their patients about whether the drug's stimulant effect could be perceived as doping or cheating. They would answer that this treatment compensate for a disability and liberate the potential. All the CAPs questioned mentioned the effects at school, principally grades, as one of the principal markers of therapeutic success. Methylphenidate allowed to meet the school system's demands so school would tolerate their patient's behavior. Some also reported having to meet a demand for academic performance.

*P7: *“*he*'*s different so society, school, don*'*t tolerate him; so we make it possible for him to be tolerated.*”

#### Effects on relationships

The adolescents reported improvements in their relationships: easier and less conflictual interactions with better integration in peer groups or calming of family conflicts.

*A2: *“*I noted that I was fighting less, I was becoming more friendly with people*”*.*

Nonetheless, the adolescents also described some negative effects on relationships, in particular a loss of spontaneity and paying more attention to others' judgments. Several even reported an experience of no longer associating with their peers, of becoming isolated.

*A15 *“*I noticed that it was cutting off my relationships with others.*”

The CAPs raised the question of the effects on relationships through the prism of the calming of family conflicts or of its effects on schooling; they argue that the medication improves the relationships with teachers.

*P7 *“*the teachers also have a representation, and as a result they say to themselves, no it*'*s not that he doesn*'*t care about anything, in fact, he*'*s different.*”

#### The changes within their sense of self

The adolescents identified either an increase or a decrease in their appetite that they associated explicitly with the treatment, with some worried about their growth, both height and weight. They also mentioned sleep problems frequently, especially insomnia. They also described a change in their thoughts, which they experienced as less intrusive, and in their imaginative life and their daydreams, which they perceived as less intense.

*A9: *“*Before, I was extremely dreamy, that is, I always had a story in my head, that I was inventing, like that … but that was before.*”

Some also observed moderation of their emotional expression. Few described their inability to perceive their emotions and reported experiences of ennui, and loss of both desire and pleasure. Some adolescents mentioned their impression that the treatment masked who they really were, that they no longer recognized themselves.

*A14: *“*I don*'*t know if it*'*s a medication or a form of torture, except for tests at school (…), the feeling of malaise that I can feel sometimes, it*'*s torture … I*'*m someone who is well connected to my feelings, but it reconfigures me.*”

Some CAPs said that they warn the patients that the treatment is going to modify their behaviors but not their personality. Most of the CAPs reported considering the adolescents' subjectivity and addressing with them the medication's effects on them personally, especially around the themes of self-esteem, self-control, and freedom.

*P6 *“*It*'*s to optimize their own abilities, but it*'*s not going to affect their temperament or their personality*”*.*

The prescribers nonetheless remain more focused on the objective markers of the therapeutic effect than of the changes of adolescents’ sense of self, in choosing to target the elements identified by neuropsychological tests and by using terms such as "correct” (P2, P5, P11),” “compensate” (P1, P2, P8), “optimize” (P3, P6),” or “boost” (P3). Finally, several CAPs mentioned "contradictory" effects associating an improvement of external functions, but a subjective deterioration reported by the teen.

*P7: That*'*s where it gets a little complicated; they see that they are working better in school, and nonetheless they feel that things are not ok and that they are no longer themselves.*

## Discussion

This qualitative study explored the lived experience of methylphenidate use among French adolescents with ADHD and CAPs.

Many of our findings were already reported by the qualitative literature from English-spoken countries. Positive outcomes, such as improvement of both family and peer relationships and better academic performance described in several studies^37,39^, are reported here by both the French adolescents and CAPs. Our findings also reported negative experiences with methylphenidate use, already described in the qualitative literature, that is the side effects^28,35,36^, and the changes in sense of self^28,34,37^ but also some negative effects on their peer relationships related with these changes in sense of self, with few adolescents even experiencing social isolation.

The issues of both the sense of self of adolescents with ADHD and how it is modified by methylphenidate is particularly emphasized in our findings. Singh^49,50^ has already described how both ADHD diagnosis and treatment impact the self-concept and self-understanding of adolescents, leading for example to negative self-attributions, while Charach et al.^34^ have reported how methylphenidate can negatively affect the adolescent's sense of self, especially due to the product's rapid effect.

A metasynthesis exploring adolescents' lived experience of ADHD found behaviors, emotions, and thoughts experienced as "things that exist outside themselves, as aspects not related to their will and intentions but rather to the ADHD (…) this experience may create a feeling of separation between the self and one’s own behaviors, thoughts, and emotions”^51^. One relevant finding in our qualitative study is in line with this feeling of separation between the self and the outside world: The French adolescents in our sample didn’t, initially, neither recognize nor accept the diagnosis of ADHD. Only externalized behaviors and the perception of others made it possible to diagnose ADHD and led to methylphenidate treatment. Some studies have demonstrated that adolescents with ADHD can have a weak self-awareness of their disorder, inaccurate perceptions of their symptoms and a low level of agency^27,52^. They often display a *positive illusory bias*, with positive ideas about their skills and competence, despite their difficulties^53^. Yet, in our study, the adolescents were aware of their externalized (ab)normal behaviors but did not consider them as pathological, as signs or symptoms of a medical condition. Moreover, some adolescents in our sample considered ADHD to be an external attribution, and other raised the issue of methylphenidate as a doping drug. We can question whether the issue is at a self-awareness and agency level or more related with social representations of ADHD in France. The adolescents of our sample were in fact mirroring a critical epistemic position denying the existence of ADHD as a biomedical entity and denouncing both the medicalization of social (ab)normal behaviors and the risk of biological empowerment^54^; while the French CAPs prescribing methylphenidate considered this prescription as the proof of their affiliation to another epistemic position—naturalistic—stating the well-established existence of ADHD as a neurodevelopmental disorder^54^. There is a substantial French literature about the reception and implementation of the concept of ADHD in France. Even if the current tendency is to integrate different models so to have a multidimensional understanding of ADHD, Bader and Mazet^55^ underlined how the French child and adolescent psychiatry culture—influenced by psychoanalytical theories, focusing on intrapsychic factors and relational dimensions, as well as on the symbolic aspects of symptoms—have shown some reticence and resistance against the implementation and use of ADHD concept since the second half of the twentieth century. Several French scholars, from medical and social sciences, have been denouncing a “scandalous” disorder^56^ or a “false epidemy”^57^. Moreover, a recent study has found major distortions of scientific consensus (95.5%) between 45 French medical theses dedicated to ADHD published between 1990 and 2018 concerning the etiology, diagnostic and therapeutic strategies^58^, the authors pointing the ideology presiding over physicians' social representations of ADHD. French CAPs prescribing methylphenidate to adolescents with ADHD should take into consideration this specific context and explore with each adolescent what are his or her representations of both ADHD and methylphenidate before initiating such treatment.

Moreover, there is also a substantial literature about the stigmatization of adolescents with ADHD. Persons with ADHD are at high risk to face stigma, prejudices, and discrimination^59^. Public’s uncertainty concerning the validity of an ADHD diagnosis, public’s skepticism toward ADHD medication and disclosure of diagnostic status as well as medication status of the individual with ADHD have been identified as variables contributing to stigma in ADHD^60^. The stigmatization affects teens substantially, adding with supplementary psychological distress and increasing the risk of at-risk behaviors, substance abuse, and the loss of social support^61^. Our findings suggest that during their encounters with their CAPs, the adolescents in our sample could not express fully neither their self/inner experience (testimonial injustice), nor their interpretations, social representations, or epistemic positions (hermeneutic injustice). These adolescents might be victims here of epistemic injustice^62^, a mechanism that amplifies their stigmatization and its harmful effects. Even more, the way that the adolescents in our sample resign themselves to the passive acceptance of this treatment echoes the cognitive bias of the labeling effect: as if some adolescents finished by conforming themselves to the exterior (and normative) gaze upon them and have integrated it into their self-concept, thus biasing their judgment of their future activities.

### Clinical implications

Our findings suggest that French CAPs prescribing methylphenidate to adolescents should address, before initiating and during the treatment, both the issues of the adolescents' own perceptions and awareness of their ADHD symptoms and their social representations and epistemic positions toward both ADHD and methylphenidate. Addressing such issues, we think, could prevent potential epistemic injustice and the amplification of stigmatization through self-stigma (internalized stigmatization) process.

### Limitations

This qualitative study has some limitations that must be considered. First, it took place in France, and caution is required in transposing our findings to other places because, as discussed above, fondings are embedded in the French sociocultural context, in general, and within the French system of child and adolescent psychiatry, in particular. Second, the population of adolescents was recruited in specialized departments of child and adolescent psychiatry, and all the psychiatrists specialized in children and adolescents. Our results therefore do not apply to adolescents receiving this treatment in other contexts of care or specialties (general practitioners, neuropediatricians). Third, subgroup analysis of adolescent boys and girls did not reveal any differences between them in relation to these results, although gender differences in both the phenomenology of ADHD and treatment effects have been described in the literature^63^. Fourth, we found no difference in the lived experience of the four adolescents who were diagnosed and started treatment only during adolescence (after the age of 12 years) compared with those diagnosed at a younger age, yet further research focusing on late diagnosis (among older adolescents and young adults) could be of interest to explore and compare aspects of our results especially regarding identity and interiority. Finally, in our study design we sought to recruit only CAPs prescribing methylphenidate, so to cross the perspectives with the adolescents treated with it. However, the practices of French CAPs vary strongly both in their acceptance of the concept of ADHD and in their use of psychostimulants as treatment. Further research should explore this diversity of thoughts and practice among French child and adolescent psychiatrists.

## Conclusion

These results of this study directly question both the roles of the self-perceptions and epistemic positions of the adolescent with ADHD in the clinical evaluation and decision-making concerning the prescription for methylphenidate. Treatment decisions should be based on external aspects but the adolescent's sensitive experience and perception plus his or her own interpretation and social representations of ADHD should be discussed during the consultations. And what about the adolescent's consent? We consider that it is impossible to be satisfied with the adolescent's passive acceptance or secondary adherence based only on exterior and normative appearances. Adolescents with psychiatric disorders should be given opportunities to be the agents of their own management and change^64^.

## Supplementary Information

Supplementary Information.

## Data Availability

The datasets analyzed during the current study are available from the corresponding author on reasonable request.
